# Effect of pH on the Poly(acrylic acid)/Poly(vinyl alcohol)/Lysozyme Complexes Formation

**DOI:** 10.3390/molecules29010208

**Published:** 2023-12-29

**Authors:** Simona Morariu, Mihaela Avadanei, Loredana Elena Nita

**Affiliations:** “Petru Poni” Institute of Macromolecular Chemistry, 41-A Grigore Ghica Voda Alley, 700487 Iasi, Romania; mavadanei@icmpp.ro (M.A.); lnazare@icmpp.ro (L.E.N.)

**Keywords:** poly(acrylic acid), poly(vinyl alcohol), lysozyme, interpolymer complexes, physical interactions

## Abstract

The interactions between poly(acrylic acid) (PAA), poly(vinyl alcohol) (PVA), and lysozyme (Lys) in an aqueous environment at pHs of 2, 4, and 7.4 were discussed considering the experimental data obtained by turbidimetry, electrokinetic and rheological measurements, and FTIR analysis. It was found that the increase in PAA amount reduces the coacervation zone by shifting the critical pH_cr1_to higher values while the critical pH_cr2_ remains unchanged. The coacervation zone extended from 3.1–4.2 to 2.9–4.7 increasing the Lys concentration from 0.2% to 0.5%. The zeta potential measurements showed that the PAA–PVA–Lys mixture in water is the most stable in the pH range of 4.5–8. Zero shear viscosity exhibited deviations from additivity at both investigated pHs, and a maximum value corresponding to a maximum hydrodynamic volume was revealed at PAA weight fractions of 0.4 and 0.5 for pHs of 4 and 7.4, respectively. The binding affinity to Lys of PAA, established by molecular dynamics simulation, was slightly higher than that of PVA. The more stable complex was PAA–Lys formed in a very acidic environment; for that, a binding affinity of −7.1 kcal/mol was determined.

## 1. Introduction

Poly(acrylic acid) (PAA) and poly(vinyl alcohol) (PVA) have gained much attention due to the possibility of developing, through their combination, materials with well-controllable properties for a wide variety of applications. A significant number of studies refer to materials based on PAA–PVA mixtures, which, due to their great versatility, can have a multitude of applications: solid polymer electrolytes [1,2], materials for the removal of pollutants from wastewater [3,4,5,6,7], hydrogels for soilless cultivation [8], food packaging applications [9], drug delivery systems [10,11], materials for various biomedical applications [12,13], etc. PAA is a weak polyelectrolyte with carboxyl groups along the main chain that dissociate as the pH increases. Up to pH = 4.5, the degree of dissociation of COOH groups from PAA chains is very low (0.03), and macromolecular chains adopt a compact globular conformation due to the hydrogen bonds and van der Waals attractive forces between groups on the PAA chain. By increasing pH above 4.5, the dissociation degree increases rapidly, reaching 1 at pH = 9, and PAA chains expand into a stretched conformation as a result of the electrostatic repulsion between the COO^−^ groups along the PAA chains [14]. PAA with a molecular weight lower than 16.5 × 10^3^ g/mol maintains the extended conformation even at low pH [15].

The investigations by isothermal titration calorimetry of the combination of PAA with PVA in aqueous solutions (2 wt/v% for PAA, 0.2 wt/v% for PVA) evidenced that no PAA–PVA complexes are formed at pH = 2, and the change in enthalpy is 0.03 kJ/mol [16]. However, the addition of PVA solution with a high concentration (5–15 wt%) increases the ionization degree of PAA for a given pH. PVA chains exhibit a coil conformation, causing a crowding effect on PAA chains, which determines the increase in local PAA concentration and, implicitly, the modification of the PAA conformation [17].

The capacity of polyelectrolytes to interact with proteins was exploited in the development of some systems used in the separation or purification of proteins [18,19,20] or some support materials for protein release [21,22,23]. The interaction between protein and polyelectrolyte can form soluble complexes, precipitates (insoluble complexes), or complex coacervates as a function of the medium condition (ionic strength, pH, protein/polyelectrolyte ratio) [24,25]. Two mechanisms are proposed for explaining the polyelectrolyte–protein interactions: (i) charge regulation, and (ii) charge anisotropy. In charge regulation theory, the polyelectrolyte attracts the counterions from the protein by columbic force, changing the pH value and the ion distribution of the environment surrounding the protein [26]. In the second mechanism, the charge anisotropy of the protein surface favors the formation of the polyelectrolyte–protein complex by electrostatic interactions as a result of the balance between short-range attraction and long-range repulsion [27].

Through the manipulation of environmental pH, various materials, including polyelectrolytes [28], DNA [29], proteins [30], lipids [31], and peptides [32] exhibit responsive self-assembly behaviors. By changing the pH, it is possible to modify the charge, solubility, conformation of molecules, and intermolecular interactions of these compounds. The use of pH modulation in self-assembly offers a simplified and efficient method to obtain functional materials with tailored properties for applications in drug delivery systems, sensors, and tissue engineering. Interest in the preparation of DNA hydrogels is growing due to their porous 3D structure, tissue-like elastic properties, and the capacity for efficient programming through nucleic acid sequences. This interest led to the development of a new preparation method besides the one that supposes the self-assembly of synthetic linear or branched DNA motifs, namely rolling circle amplification (RCA) [33,34]. In the synthesis of DNA hydrogels, RCA is used to amplify DNA, which then forms a 3D hydrogel network through various cross-linking strategies, including entanglement of DNA chains, multi-primed chain amplification, hybridization between DNA chains, and hybridization with functional moieties [35,36].

Among proteins, lysozyme (Lys) has attracted attention, on the one hand, due to its antimicrobial activity and, on the other hand, its availability from a wide range of natural resources (tears, saliva, mucus, milk) [37]. Discovering it “quite by chance” in 1922, Fleming for the first time proved the antimicrobial activity of Lys [38]. Lys can hydrolyze the peptidoglycan linkages of bacterial cell walls, making it an important natural compound with antibacterial activity. It was also proven that Lys has tumor-inhibitory activity and that it also improves the efficiency of chemotherapeutic treatments [39]. Lys is a protein having a positive charge at a pH lower than its isoelectric point. Positive charges of 10 (19 positive and 9 negative) and 6 (17 positive and 11 negative) at pHs of 4.5 and 9, respectively, were estimated [40].

Romanini et al. [41] found that the PAA–Lys complexes are insoluble at low pH, with a value of polymer per protein mol around 0.003–0.001. The Lys incorporation into PAA microgels takes place in two steps: (i) in the first stage, the Lys-microgel shell is formed with rapid microgel deswelling without Lys diffusion into the microgel core; (ii) in the second stage, the swelling of the microgel is negligible and Lys starts to diffuse into the microgel core [42]. The binding of Lys to PAA is exothermic and pH-dependent, with the binding stoichiometry increasing with increasing pH [43].

The capacity of Lys to form strong interactions with anionic polyelectrolytes, leading to insoluble complexes in a specific pH range, was used in protein recovery from aqueous solutions by precipitation [44]. The antimicrobial properties of Lys were exploited in the tailoring of packaging materials based on both PAA [45] and PVA [46]. The interactions between PVA and Lys do not affect the higher-order structure of Lys, and even more, PVA protects protein stability by being able to be used as an additive [47]. A wound dressing with excellent biocompatibility and activity in wound healing was developed using vanillin as a crosslinker between PVA and Lys [48]. Although a large number of investigations have been attributed to PAA–Lys and PVA–Lys complexes, the complex containing the three components (PVA, PAA and Lys) has been little investigated. Amariei and coworkers had prepared the electrospun wound dressings from blends of PAA and PVA with antimicrobial surfaces by incorporating Lys [49]. The use of Lys has shown two limitations: (i) lower antimicrobial activity compared with other peptides, and (ii) the rapid release of adsorbed Lys at physiological pH. The systems based on PAA, PVA, and Lys that could be used for ocular drug delivery were developed by incorporating chitosan-poly (acrylic acid) nanoparticles into polyvinyl alcohol (PVA) hydrogels [50]. The chitosan-poly (acrylic acid) particle diameter was significantly reduced in the solutions containing Lys due to the hydrolyzation of the chitosan from the surface of the nanoparticles. The samples with higher amounts of Lys exhibited an increase in particle diameter after 24 h due to, on the one hand, the degraded particles which swelled over time, or, on the other hand, the electrostatic interactions between PAA and Lys [50]. The combination of the biocompatibility of PAA and PVA, the pH sensitivity of PAA, and the antimicrobial properties of Lys leads to a complex material for coating titanium implants. The PAA–PVA–Lys complex may alter the surface properties of the titanium implant, improving its integration with surrounding tissues and promoting better osseointegration [51].

The designing and tailoring of new materials based on PAA, PVA, and Lys is essential to knowing and deeper understanding the formation mechanism of the PAA–PVA–Lys complexes at various pH values. The unique properties of each component contribute to the versatility and potential utility of the composite in various applications. Additionally, the interactions within the PAA–PVA–Lys complex with varying pH values enable us to optimize its stability, solubility, and bioactivity and to design new materials for a wide range of biomedical applications, including drug delivery platforms capable of controlled release, tissue engineering scaffolds with tailored properties, and antibacterial coatings that combat microbial growth.

The present study aims to provide new insights into how pH can modulate the interactions and properties of PAA–PVA–Lys complexes, potentially enabling the design of more efficient drug delivery systems or other applications that require pH-responsive behavior. The interactions between the three components were discussed, considering the experimental data obtained by turbidimetry, rheology, electrokinetic measurements, and FTIR analysis. The interactions involved in the complex formation were also investigated using in silico approaches in order to finally identify the optimal pH for protein binding in the complex.

## 2. Results and Discussion

**Figure 1:**
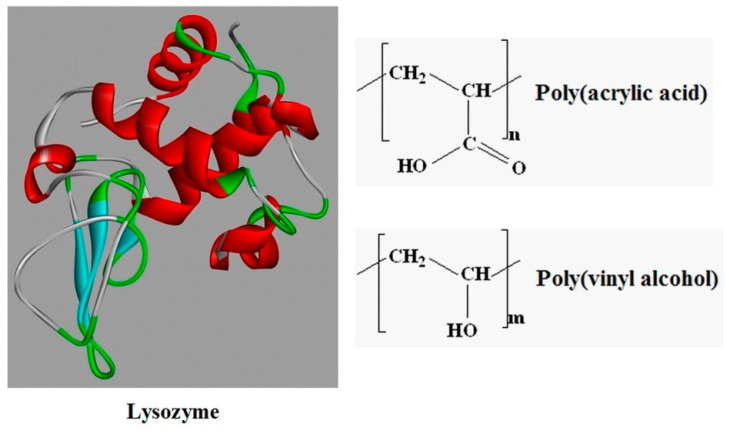
The structures of Lysozyme (from https://www.rcsb.org (accessed on 3 February 2022)), poly(acrylic acid), and poly(vinyl alcohol).

The effect of pH changes on the development of interactions in systems containing PAA, PVA, and Lys in water is discussed using both experimental and theoretical methods. The structures of Lys and the two polymers are shown in Figure 1. Human lysozyme (the type used in our study) is a natural protein that consists of one chain of polypeptide with 130 amino acid residues and a molecular mass of about 14.7 × 10^3^ g/mol [52]. It is positively charged and has a high isoelectric point of 10.5–11 [53]. PAA is a weak anionic polyelectrolyte with a pKa of 4.5. The groups COOH are partially undissociated below a pH of 4.5, and the dissociation degree of carboxyl groups increases with increasing pH value [54]. PVA is an uncharged polymer insensitive to changes in pH.

### 2.1. Interactions Investigation by Turbidimetry

**Figure 2:**
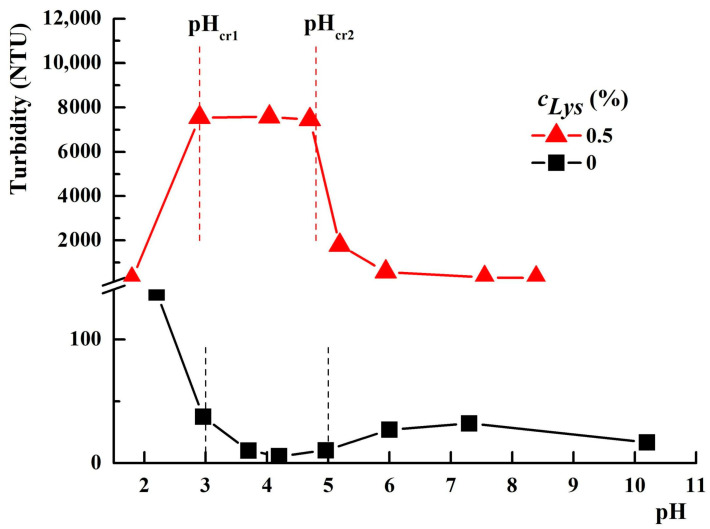
The variation of the turbidity of PAA–PVA mixtures in water in the absence/presence of Lys (*w_PAA_* = 0.7, *c_p_* = 3%).

Nurkeeva and coworkers [55] evidenced three domains in the evolution of the turbidity of PAA–PVA equimolar aqueous solutions as a function of pH, depending on the nature of the complexes formed: (i) at a pH lower than a critical value (pH_cr1_) between 2.75 and 3, the interpolymer complexes with hydrophobic properties are formed and the aggregation starts; (ii) at a pH between pH_cr1_ and 5 (pH_cr2_), the PAA–PVA hydrophilic interpolymer complexes are formed; (iii) at a pH higher than pH_cr2_, the interactions between the polymer chains with the same structure are intensified, leading to soluble complexes. These three domains were also identified by us, as shown in Figure 2, for the PAA–PVA mixture in water in the absence of protein, with a weight fraction of PAA *w_PAA_* = 0.7 and a polymer concentration *c_p_* = 3%.

By decreasing the pH value below about 3, the formation of hydrophobic interpolymer complexes determines the increase in turbidity. At pH values between 3 and 5, the turbidity decreases due to the formation of hydrophilic interpolymer complexes. Above pH = 5, a slight increase in turbidity is observed due to the formation of some less hydrophilic complexes.

**Figure 3:**
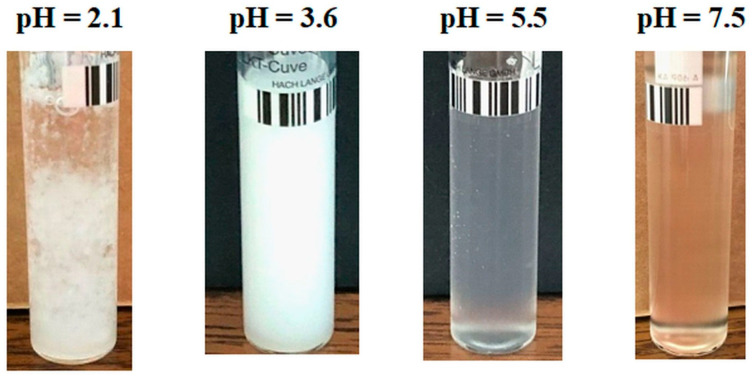
Image of samples with *w_PAA_* = 0.7, *c_p_* = 3%, and *c_Lys_* = 0.3% at various pH conditions.

**Figure 4:**
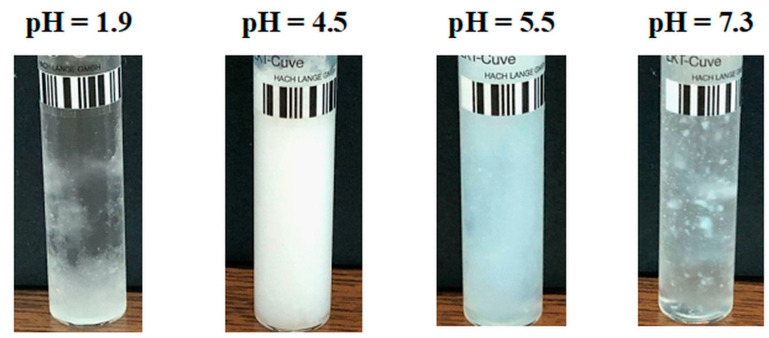
Image of samples with *w_PAA_* = 0.7, *c_p_* = 3%, and *c_Lys_* = 0.6% at various pH conditions.

An evolution in the mirror of turbidity with pH, compared to a PAA–PVA system free of Lys, was observed for the system containing 0.5% Lys (Figure 2). Thereby, in the turbidity curve of PAA–PVA–Lys aqueous solution, the following domains can be delimited: (i) at pH below pH_cr1_ (about 2.9), the turbidity decreases suddenly and the separation in two phases occurs (Figure 3—pH = 2.1 and Figure 4—pH = 1.9); (ii) at pH_cr1_ (2.9) < pH < pH_cr2_ (4.8), the turbidity reaches a plateau at about 7500 NTU and a liquid coacervate phase is formed (Figure 3—pH = 3.6 and Figure 4—pH = 4.5); (iii) at high pH (>pH_cr2_), the turbidity decreases.

For low Lys concentrations, the formed complexes are soluble in water, and a clear system is observed (Figure 3; pH = 5.5 and pH = 7.5). The increase in Lys concentration increases the hydrophobicity of the complexes formed at a basic pH. Thereby, for the sample with *w_PAA_* = 0.7, *c_p_* = 3%, and *c_Lys_* = 0.6%, a precipitate was observed at pH = 7.3 (Figure 4).

**Figure 5:**
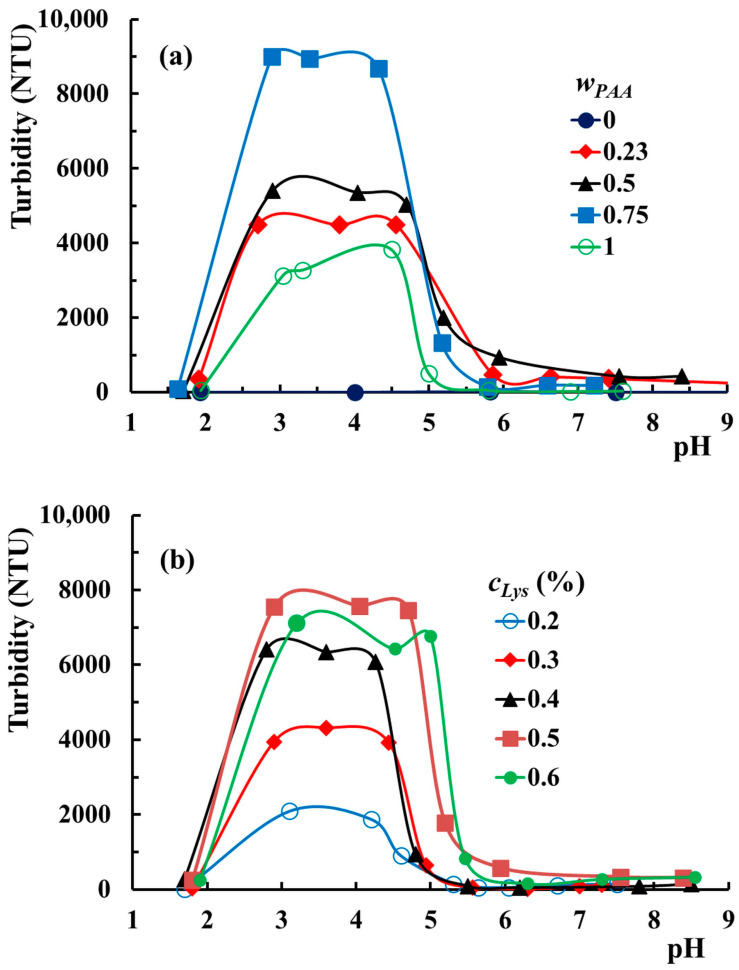
The evolution of the turbidity versus pH for PAA–PVA mixtures in Lys solution with (**a**) 0.5% Lys, *c_p_* = 3%, and various *w_PAA_*; (**b**) *w_PAA_* = 0.7, *c_p_* = 3%, and various *c_Lys_* at room temperature.

In order to understand the interactions established as a function of pH and the role of each component in the observed phenomena, the effect of *w_PAA_* and *c_Lys_* on the pH intervals corresponding to each phase observed above was studied. The effect of the PAA amount on the turbidimetric curves is illustrated in Figure 5a.

The turbidity of the PVA solution containing 0.5% Lys is very low (lower than 10 NTU) and independent of the medium pH. The addition of PAA makes the system sensitive to pH changes. All aqueous mixtures containing PAA and Lys (including those without PVA) show three domains characteristic of the PAA–PVA–Lys mixture in water. The increase in PAA in mixtures moves pH_cr1_ to slightly higher values. Thereby, pH_cr1_ increases from 2.9 for *w*_PAA_ = 0.5 to 3.3 for *w*_PAA_ = 1. The pH_cr2_ value does not appear to be affected by the addition of PAA. Nurkeeva et al. [55] reported that pH_cr1_ for systems PAA–PVA without lysozyme with polymer concentrations between 10^−2^ mol/L and 10^−1^ mol/L and PAA/PVA = 1/1 was within the range of 2.75–3. In our study, the polymer concentration expressed in mol/L is around 10^−4^ mol/L, and pH_cr1_ would be expected to be lower than those reported by Nurkeeva and coworkers. The higher values obtained in our investigations at lower polymer concentrations prove that the complexation ability of PVA is higher in the presence of Lys. Other polymers containing hydroxyl groups, such as poly(2-hydroxyethyl acrylate) and poly(2-hydroxyethyl vinyl ether), exhibit similar complexing abilities, showing pH_cr1_ between 2.6–2.8 and 2.5–2.9, respectively [56,57]. On the contrary, poly(vinylpyrrolidone) and poly(ethylene oxide) proved higher ability to complex in the mixture with PAA, showing pH_cr1_ values in the range 4.5–5.2 and 2.8–3.7, respectively [58,59]. In Figure 5b, the effect of the Lys concentration on the mixtures’ turbidities is presented. The three ranges are also evidenced in the variation of turbidity at different *c_Lys_*. The turbidity of the liquid coacervate phase increases by increasing *c_Lys_* up to 0.5%, and a further addition of Lys does not significantly change this parameter. The increase in *c_Lys_* determines the slight widening of the pH range corresponding to the liquid coacervate phase. Thereby, the pH_cr1_–pH_cr2_ range extends from 3.1–4.2 for *c_Lys_* = 0.2% to 2.9–4.7 for *c_Lys_* = 0.5%. The mixture with *c_Lys_* = 0.6% shows a large pH domain for the liquid coacervate phase but shifted to higher values (3.2–5).

### 2.2. Complexes Investigation by FTIR

**Figure 6:**
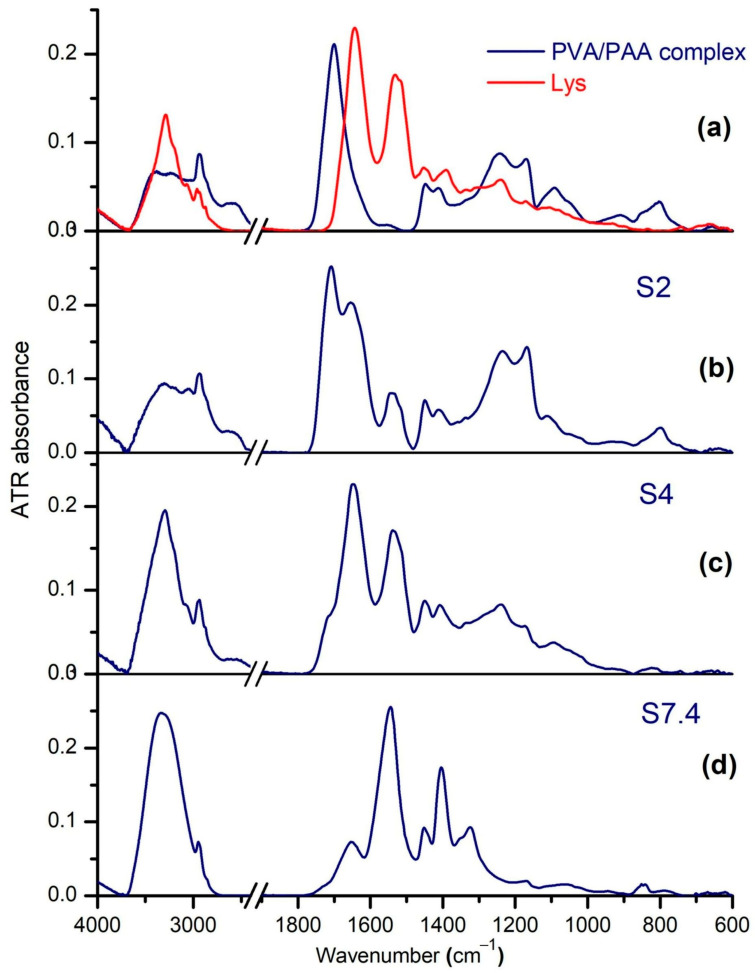
The ATR-FTIR spectra of (**a**) PVA–PAA complex and Lys, and of PAA–PVA–Lys complex at (**b**) pH = 2 (S2), (**c**) pH = 4 (S4), and (**d**) pH = 7.4 (S7.4).

Figure 6a–d shows the ATR-FTIR spectra of the PAA–PVA solid complex, Lys, and of the complexes obtained from the mixtures PAA–PVA–Lys in water with *w_PAA_* = 0.5, *c_Lys_* = 0.6%, and *c_p_* = 2.7% at various pH values. In the FTIR analysis part, the PAA–PVA–Lys mixtures have been named S2, S4, and S7.4 for pHs of 2, 4, and 7.4, respectively, in order to make it easier to follow the explanations. For the pure PVA–PAA blend (Figure 6a), the ν(OH•••) vibrations of PAA at 3080 cm^−1^ and of PVA at 3296 cm^−1^ are replaced by two peaks at 3247 and 3407 cm^−1^, confirming the strong intermolecular connections between them. Because of the high amount of PAA, the carbonyl stretching of PAA has a small shift, from 1700 to 1701 cm^−1^. The crystallinity sensitive band of PVA at 1142 cm^−1^ disappeared, so in this compact network, PVA is in the amorphous phase. The polymeric signals in the S2, S4, and S7.4 complexes (Figure 6c,d) are perturbed due to the different states of ionization of carboxylate groups, of the amino acid residues of Lys, and of the direct interaction with the protein.

PVA in the amorphous phase is barely visible in S2 and S4 at ≅1105 cm^−1^ (ν(C–O) + δOH) [60], which is slightly blueshifted from 1085 cm^−1^ in PAA–PVA. The IR spectrum of S7.4 contains some peaks connected to the gauche and trans conformers in small amorphous domains, such as at 1095 cm^−1^ (ν(C–C)_C-C-G_), 1066 and 1049 cm^−1^ (ν(C–C)_C-C-T_), 1019 (ν(C–OH) and 852 cm^−1^ (ω(CH)) [60]. On the other side, PAA is in acid form in S2 and S4, with ν(C=O) blueshifted as compared to its position in pure PAA: at 1708 cm^−1^ in S2 and at 1716 cm^−1^ (sh) in S4.

The blueshift of ν(C=O) in S2 has been analyzed in terms of spectral decomposition into its components (Figure S1 in Supplementary Materials). The sub-bands at 1740, 1727, and 1704 cm^−1^ can be assigned to free acid groups, to those involved in intermolecular C=O•••HO-hydrogen bonding between PAA and Lys, and to those of the cyclic dimers in PAA alone, respectively. The sub-band at 1727 cm^−1^ has been developed in the blends formed at pH = 2 and pH = 4 at the expense of the self-associated COOH at 1704 cm^−1^. In addition, the strength of the PAA–Lys interactions is weaker than the hydrogen bonding in the dimers of PAA. Still, the ν(C=O) absorption in S4 is of low intensity, assisted by a flattening of the strong intermolecular hydrogen bonds at 2600 cm^−1^, and such a drastic decrease in intensity may be explained by a change in the strength of hydrogen bonding. Curve-fitting with good accuracy included a new band around 1690 cm^−1^ (Figure 6b) that can belong to carboxyl groups with very strong H-bonds.

S7.4 contains the ionized form of PAA, recognized by the vibrations of carboxylate COO^−^ at 1651 cm^−1^ and 1404 cm^−1^. As a result, the S7.4 complex lacks the intermolecular ν(OH•••) at 2567 cm^−1^ between the COOH groups. The Amide I band of native Lys is observed at 1646 cm^−1^, as a superposition of the two dominant secondary structures: the α-helix at 1650 cm^−1^ and random coils at 1640 cm^−1^ [61]. The blueshift with 10 cm^−1^ of Amide I in S2, S4, and S7.4 suggests a re-organization of some structural components of Lys induced by interaction with the matrix.

The spectral decomposition and the bands assignment synthesized in Table 1 led to the detection of changes experienced by Lys in the three blends.

**Figure 7:**
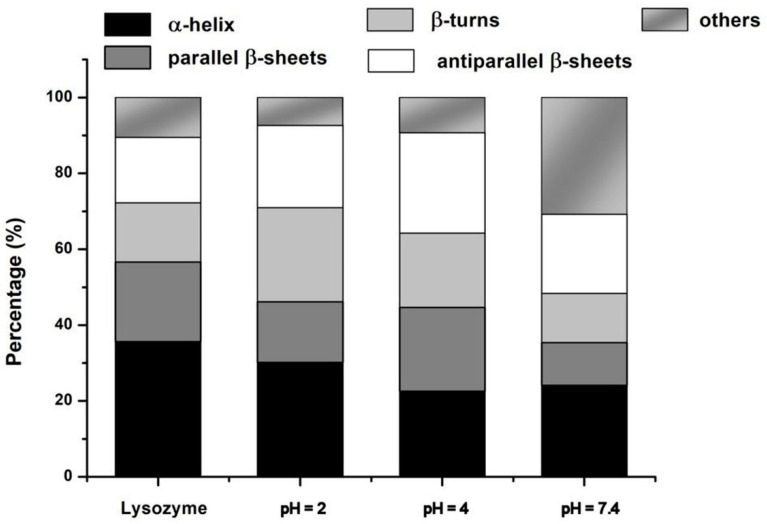
Histogram of the secondary structures fraction of Lys in pure form and in S2, S4, and S7.4 complexes, calculated from spectral decomposition of the Amide I band.

The histogram of the secondary structure fraction presented in Figure 7 shows that the formation of Lys–PAA–PVA complexes did not significantly affect the content of regular α-helices, excepting that at pH = 7.4.

At low pH and in the context of a strong interpolymer complex, it appears that some of the unordered polypeptide chains were arranging into regular structures such as intermolecular (antiparallel) β–sheets, as identified by the enhancement of the sub-band around 1680 cm^−1^ and the lower intensity of the band around 1645 cm^−1^. The increased content of intermolecular β–sheets and decreasing of the regular β–sheets must be connected with the partial defolded state of Lys in acidic conditions [62], which enhances the protein interactions. The content of β–turns apparently increases as well. One may analyze this fact as new electrostatic interactions between the matrix and the polar and charged groups of β–turns. These changes in the Lys components were connected to the disappearance of the weak H-bonded ν(OH•••) groups at 3398 cm^−1^ from the native PAA–PVA blend and the decrease in the band centered on 2600 cm^−1^, which means that some of the hydrogen bonds made between carboxyl and hydroxyl groups in the matrix were transferred to interactions with the protein.

The spectrum of S4 is similar to that of Lys and shows the same ν(NH) at 3296 cm^−1^; it is supposed that most of the protein is very regular and is interacting with the matrix at the level of the unordered chains. We can envisage a hypercomplex where Lys is a junction point between PAA and PVA. The left wing of Amide I in S7.4 is clearly intensified in comparison with native Lys and with S2. Here, there are contributions from intermolecular (antiparallel) β–sheets and, probably, weakly H–bonded carboxylates from PAA.

### 2.3. Zeta Potential Data

**Figure 8:**
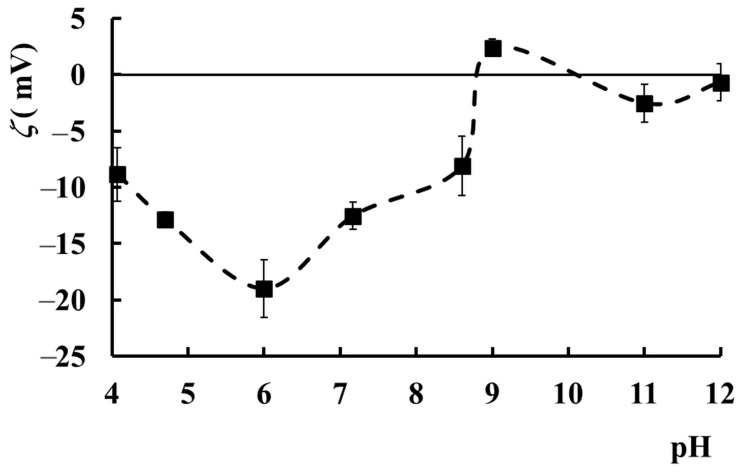
The variation of *ζ* as a function of pH value for the sample with *w_PAA_* = 0.5, *c_p_* = 2.5%, and *c_Lys_* = 0.2%. Error bars represent the average values ± standard deviation.

The zeta potential (*ζ*), which is a measure of the surface charge of a particle in a liquid medium and an indicator of its stability, varies with pH for PAA–PVA–Lys complexes in an aqueous environment (Figure 8). Lys is a positively charged protein, and this charge decreases as the pH increases, while PAA becomes more negatively charged as the pH increases due to the ionization of the carboxyl groups. The *ζ* values are mainly due to the charge of the complexes formed between Lys and PAA. PVA may not have a significant interaction with Lys or PAA due to its neutral nature. The sample with *w_PAA_* = 0.5, *c_p_* = 2.5%, and *c_Lys_* = 0.2% is in an incipient instability phase at pH values between 4 and 8 (*ζ* from −10 mV to −19 mV), showing a minimum value at pH = 6. The *ζ* value decreases from −10 mV to −19 mV with pH increasing from 4 to 6 due to the interplay of several factors: (i) the ionization of carboxylic groups in PAA chains, which determines the decrease in the negative zeta potential; (ii) the weakening of the electrostatic interactions between ionized PAA chains (negative charge) and Lys, whose positive charge decreases by increasing pH, determining an increase in the net negative charge on the complex; (iii) the conformational changes as a function of pH can alter the charge distribution of the complex surface, leading to variations in the zeta potential.

At pH = 6, there is probably a balance between protonation and deprotonation of the carboxyl groups on PAA and a balance between the positive charge of Lys and the negative charge of PAA, resulting in a minimum zeta potential. This equilibrium determines the formation of a more stable complex due to diminished repulsion forces. The further increase in pH above 6 determines the decrease in the *ζ* value as a result of the masking of COO^−^ from PAA by their electrostatic binding with the NH_3_^+^ ions from Lys. However, the electrostatic interactions between the two molecules are weaker than at an acidic pH because the lysozyme is less positively charged. For pH > 9, *ζ* acquires values very close to zero, and the system becomes unstable. From zeta potential variation with pH, it can be observed that the PAA–PVA–Lys mixture in water (*w_PAA_* = 0.5, *c_p_* = 2.5%, and *c_Lys_* = 0.2%) is the most stable in the pH range of 4.5–8, where *ζ* values have the lowest negative values.

### 2.4. PAA–PVA Interactions Investigation by Rheological Measurements

The interactions between Lys, PAA, and PVA are complex and depend on a number of factors, including the pH, ionic strength, concentration of the components, and the ratio between components. The rheological investigations aimed to find the effect of pH and the ratio between PAA and PVA at a constant concentration of Lys on the interactions in the system. The turbidimetric investigation evidenced that, in the approximate pH range of 2.5–4.5, the systems form a turbid phase (coacervated phase) and, at the basic pH and low concentration of Lys (*c_Lys_* lower than about 0.4%), the systems are more stable (Figure 3 and Figure 4). Considering the observations from the turbidimetric study, the rheological investigations were carried out on PAA–PVA–Lys mixtures with *c_p_* = 2.5%, *c_Lys_* = 0.2%, and various weight fractions of PAA at pHs of 4 and 7.5. The rheological data obtained by the steady shear experiments were fitted with the Carreau–Yasuda model (Equation (1)) in order to determine the zero shear viscosity ($\eta_{0}$) values [63]:

(1) ηγ˙=η0/1+λ·γ˙x1−nx

where λ is a characteristic time and *n* is the slope in the power law region.

In Figure S2 (Supplementary Materials), the variation of the apparent viscosity, η, as a function of shear rate, $\dot{\gamma }$, for the PAA–PVA mixture in Lys aqueous solution at pHs of 4 and 7.5 is exemplified. The values $\eta_{0}$ of the mixtures at the two pHs (4 and 7.5) are shown in Table 2. It can be observed that the increase in pH determines the viscosity increase, excluding the PVA–Lys mixture free of PAA, which shows similar viscosities. The zero shear viscosity increases from 10.6 mPa·s to 122.6 mPa·s at pH = 4 and from 10.9 mPa·s to 461.1 mPa·s at pH = 7.4.

In an acidic condition, the samples with a lower PAA content (*w_PAA_* < 0.9) have a power law index, *n*, very close to unity, indicating flow behavior very close to the Newtonian one. The flow behavior acquires a more pronounced pseudoplastic character (*n* diminishes) for the samples with *w_PAA_* = 0.9 and for the sample free of PVA. In the basic medium, only the mixture free of PAA shows a Newtonian behavior, while the addition of PAA changes the rheological properties of samples, giving them a slightly pseudoplastic character.

For an ideal mixture of polymer solutions free of interactions, the additivity rule can be theoretically calculated with the following relationship [64]:

(2) $$\log\eta_{0}=w_{PAA}\cdot \log\eta_{0PAA}+w_{PVA}\cdot \log\eta_{0PVA}$$

where *w_PAA_* and *w_PVA_* represent the weight fractions of PAA and PVA, respectively; $\eta_{0PAA}\;$and $\eta_{0PVA}$ are zero shear viscosities of PAA and PVA, respectively.

**Figure 9:**
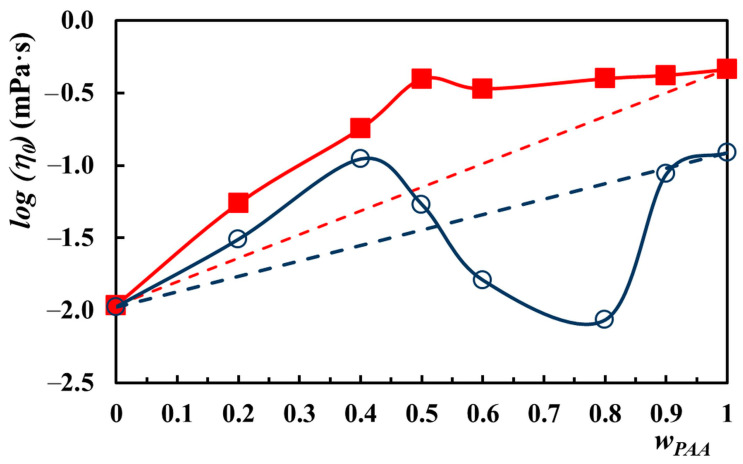
Logarithm of zero shear viscosity as a function of *w_PAA_* for PAA–PVA mixture (*c_p_* = 2.5%) in 0.2% Lys aqueous solution at pH = 4 (empty symbols) and pH = 7.4 (filled symbols); the dotted lines represent the additive rule calculated with Equation (2).

Figure 9 shows the deviation from additivity of the zero shear viscosity of PAA–PVA mixtures in Lys aqueous solution at pHs of 4 and 7.4. For *w_PAA_* below 0.52, a positive deviation was found at acidic pH as a result of the interaction between the two polymers, which leads to an increase in hydrodynamic volume. By increasing *w_PAA_* above 0.52, the deviation becomes negative, and the interactions between PAA and PVA decrease the hydrodynamic volume. The zero shear viscosity obeys the additive rule for very high PAA content (*w_PAA_* > 0.90). In the basic medium, the zero shear viscosity exhibits a positive deviation regardless of the PAA content in the mixture.

A maximum value of $\eta_{0}$, higher than additivity and explained by the establishment of a maximum number of hydrogen bonds between the two polymers, was identified in the variation with *w_PAA_* for both pHs. This maximum corresponds to *w_PAA_* of 0.4 and 0.5 for pHs of 4 and 7.4, respectively. A similar maximum at *w_PAA_* of about 0.5 was observed by Li and Hsieh [65] for the PAA–PVA mixture in water with a polymer concentration of 6% and M_PAA_ = 450 × 10^3^ g/mol and M_PVA_ = 124 × 10^3^–186 × 10^3^ g/mol. The viscosities reported by Li and Hsieh were over the additivity curve and much larger due to the stronger and abundant hydrogen bonds established between COOH and OH groups in PAA and PVA [65]. However, the maximum observed by us at pH = 7.4 corresponds to a COOH/OH ratio of 0.61, close to the one reported by Li and Hsieh of 0.69. At pH = 4, the maximum shifts to a lower value of the COOH/OH ratio, namely at 0.37.

Under acidic conditions, the lysozyme may undergo conformational changes, resulting in the unfolding of the molecule and the exposure of hydrophobic regions. Thereby, in acidic pH conditions, if the sample contains an excess of PVA, this neutral polymer can undergo physical entanglement with lysozyme, leading to the formation of a more extended and bulky structure. At pH 4 and a low PAA–PVA ratio, the combination of the physical entanglement between lysozyme and PVA with the electrostatic interactions between PAA and lysozyme can lead to a higher hydrodynamic volume of the complex than would be expected from simple additivity. In acidic conditions and with a higher PAA–PVA ratio (PAA exceeds PVA), stronger interactions with lysozyme can result, leading to more compact complexes and further reducing the hydrodynamic volume below the additive behavior.

The combination of several factors could explain the increase in hydrodynamic volume of the PAA–PVA–Lys complex above the additivity curve, regardless of the PAA/PVA ratio at pH 7.4 (Figure 9). The electrostatic interactions between positive-charged Lys and negative-charged PAA in basic conditions and the steric hindrance that can restrict the conformational flexibility of the complex could be responsible for the increase in hydrodynamic volume. In addition, the hydrophilicity of PAA and PVA determines an increase in the hydrophilicity of the PAA–PVA–Lys complex, leading to a larger hydrodynamic volume compared to the individual components.

### 2.5. Lys–PAA and Lys–PVA Interactions Investigation by Molecular Dynamics Simulation

**Figure 10:**
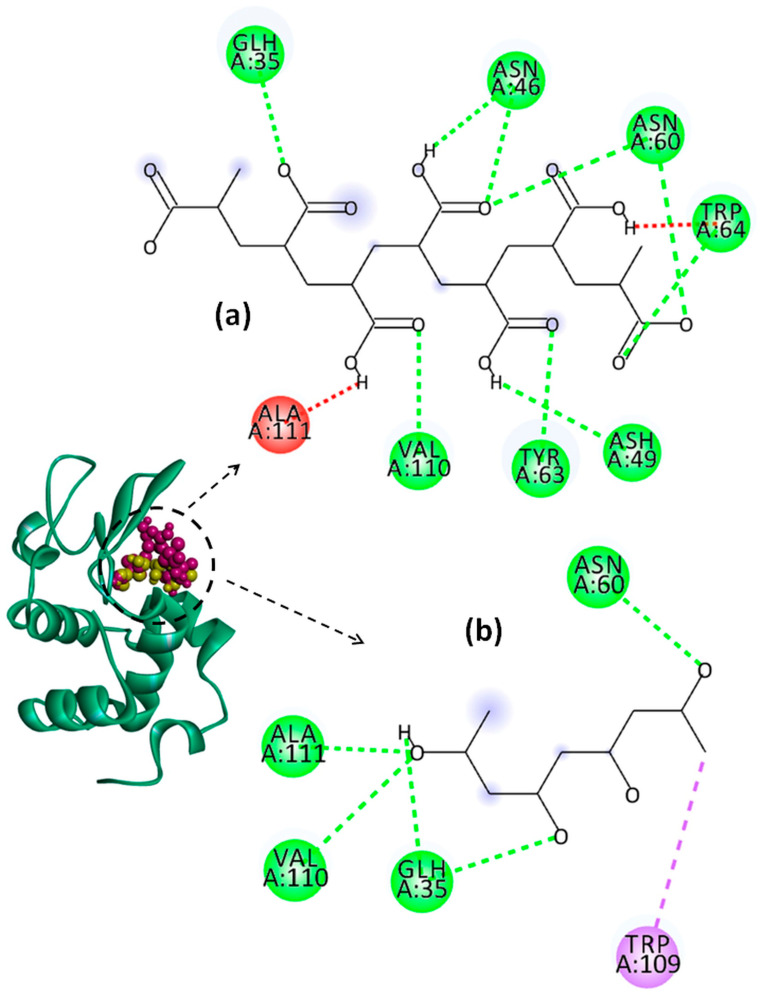
Representation of Lys chain (teal ribbon chain) with PVA (yellow balls chain) and PAA (violet balls chain); 2D diagrams of docked poses of PAA (**a**) and PVA (**b**) with Lys at pH = 2: green—classical hydrogen bond; red—unfavorable donor–donor; violet—pi–sigma. The blue halos represent the solvent–accessible surface.

**Figure 11:**
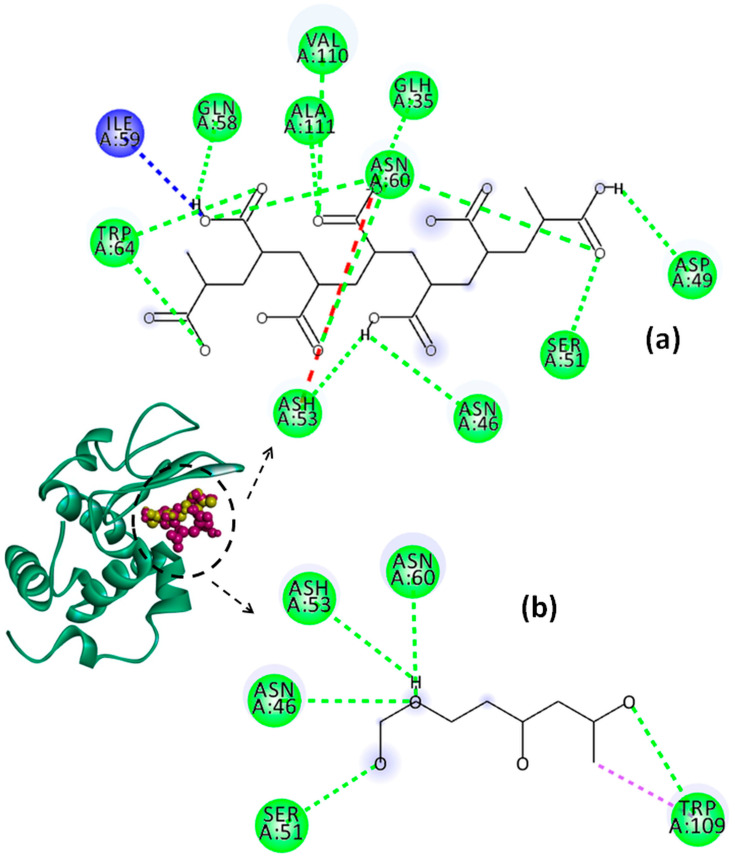
Representation of Lys chain (teal ribbon chain) with PVA (yellow balls chain) and PAA (violet balls chain); 2D diagrams of docked poses of PAA (**a**) and PVA (**b**) with Lys at pH = 4: green—classical hydrogen bond; dark blue—carbon hydrogen bond; red—unfavorable negative–negative; violet—pi–sigma. The blue halos represent the solvent–accessible surface.

**Figure 12:**
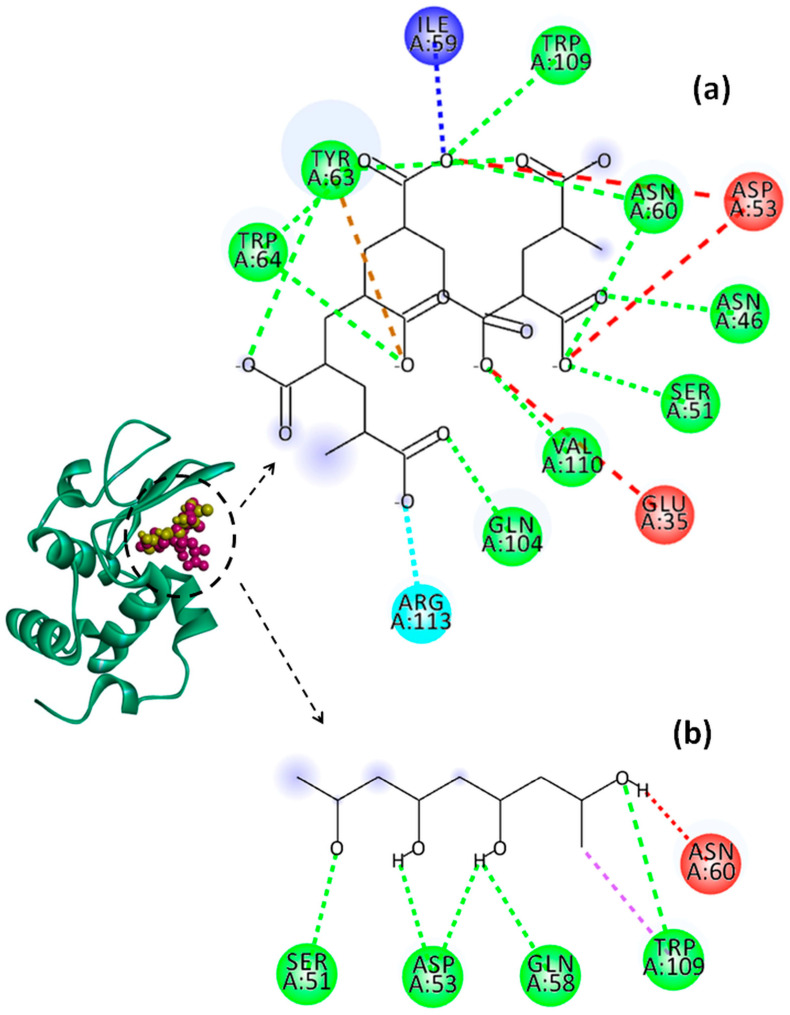
Representation of Lys chain (teal ribbon chain) with PVA (yellow balls chain) and PAA (violet balls chain); 2D diagrams of docked poses of PAA (**a**) and PVA (**b**) with Lys at pH = 7.4: green—classical hydrogen bond; dark blue—carbon hydrogen bond; light blue—salt bridge; brown—pi–anion; red—unfavorable negative–negative; violet—pi–sigma. The blue halos represent the solvent–accessible surface.

The effect of pH on the binding energy and on the types of interactions established between the system components was predicted by a molecular docking study. In this regard, numerous software packages, such as PyRx-Python Prescription 0.8, Open Babel GUI 2.4.1, AutoDock Vina 1.1.2, etc., were used. The active sites of Lys identified with the CASTp online server are shown in Table 3. The 3D structures of the most stable Lys–PAA–PVA conformers and the 2D diagrams of the interactions in acidic and basic conditions are provided in Figure 10, Figure 11 and Figure 12. In addition, for clarity, the details concerning the active sites of Lys involved in the binding interactions with PAA and PVA are presented in Table 4 and Table 5.

The interactions established between Lys and the polymer chains are mainly electrostatic interactions due to the positive charge of Lys and the negative charge of PAA [66]. In addition, non-electrostatic interactions, such as hydrophobic interactions and hydrogen bonds, are possible to form between polypeptide and polymer chains [67].

The values of binding affinity give an indication of the binding capacity of the ligand to the protein. Thereby, a more negative binding affinity indicates better binding. In our study, the binding affinities have a negative sign, which means that Lys and the investigated polymers bind spontaneously without consuming energy. The PAA–Lys interactions are stronger than those in the PVA–Lys complex, resulting in a slightly higher binding affinity (more negative). The binding affinity of PAA decreases slightly from −7.1 kcal/mol to −6.4 kcal/mol by increasing the pH from 2 to 7.4. The binding affinity of the PAA changes due to the charge distribution and conformational changes of both Lys and PAA by modifying the pH value. At pH = 2, the interactions between PAA and Lys include nine strong hydrogen bonds with bond distances lower than 2.60 Å. By increasing the pH value to 4, the number of hydrogen bonds increases to 10, but these become weaker, most showing bond distances greater than 2.5 Å. By increasing pH above 2, the conventional hydrogen bonds are accompanied by a non-conventional hydrogen bond (carbon hydrogen bond) between the ILE59 amino acid of Lys and the C=O group of PAA. Furthermore, at pHs 4 and 7.4, the repulsive forces between negatively charged residues on Lys and PAA start to develop. However, the difference between the binding affinities of PAA at the investigated pH values is not significant.

PVA shows similar values of binding affinity of about −5.3 kcal/mol at the three investigated pH values. Thereby, for PVA–Lys complexes, a hydrophobic interaction of type pi–sigma at all pH values between TRP109 and PVA was evidenced. In Lys–PAA–PVA complexes, the interactions between PAA and PVA can affect the interactions with Lys. Wei et al. [68] found that the binding energy between PAA and PVA decreases with the increase in the PAA amount in the blend. The interactions between the PVA chains are stronger than those between the PAA chains, and the complexes can be ordered by strength as follows: PVA–PVA > PVA–PAA > PAA–PAA. The higher affinity of PAA to interact with Lys, regardless of pH (proved in the present paper), and the greater stability of the PAA–PVA complex reported previously by Wei and coworkers allow us to conclude that PAA could be a bridging ligand between PVA and Lys in the PAA–PVA–Lys complexes.

## 3. Experimental Section

### 3.1. Materials

Poly(acrylic acid) (PAA) (Mw = 450 × 10^3^ g/mol), poly(vinyl alcohol) (PVA) (Mw = 166 × 10^3^ g/mol), and Human Milk Lysozyme lyophilized powder (Lys) (with higher than 10^5^ units/mg protein) were purchased from Sigma-Aldrich Co. (Taufkirchen, Munich, Germany) and used without further purification. The molecular weight of Lys considered in the calculation was 14.7 × 10^3^ g/mol [53]. The reagents such as hydrochloric acid (35%) (HCl) and sodium hydroxide (NaOH) were also supplied by Sigma-Aldrich Co.

### 3.2. Samples Preparation

Lys and PAA solutions with concentrations of 1.5% and 5%, respectively, were prepared in deionized water by stirring for 8 h at room temperature. A 5% PVA solution was obtained by dissolving the polymer in deionized water at 80 °C under vigorous stirring for 8 h and kept overnight at room temperature to reach the equilibrium state. To obtain the PAA–PVA–Lys mixtures, various amounts of PVA and PAA solutions were mixed to obtain samples with different weight fractions of PAA (*w*_PAA_) in the PVA–PAA mixture. Then, a certain amount of Lys solution was added to the polymer mixtures so that the final polymer concentrations were 2.5%, 2.7%, or 3%, and that of Lys was in the range of 0.2–0.6%. The pH values, measured with an AD12 pH meter (Adwa Instruments, Szeged, Hungary), were adjusted by adding drop by drop of NaOH or HCl solutions (0.1 or 1 M). The concentrations of the initial solutions and of the components in the final mixtures are expressed as weight percentages.

### 3.3. Analysis Methods

*Turbidity* (expressed as Nephelometric Turbidity Units, NTU) was determined at 25 °C by using an HACH 2100AN turbidimeter (Hach Co., Loveland, CO, USA), which has a tungsten lamp and a filter assembly that limits the wavelength in the range of 400–680 nm. Before each measurement, the turbidimeter was calibrated with Stablcal^®^ Stabilized Formazin Standards, 0–7500 NTU. The turbidimeter is equipped with four detectors: a 90-degree detector, a forward scatter light detector, a back scatter detector, and a transmitted light detector. Selecting all detectors (Ratio mode), the turbidimeter measures the turbidity up to 10,000 NTU, and, by activating Signal Averaging mode, the values given by the device are an average of 10 measurements. The turbidimeter provides the measured values with an accuracy of ±2% for 0–1000 NTU, ±5% for 1000–4000 NTU, and ±10% for 4000–10,000 NTU.

*Zeta potential* (*ζ*) measurements were performed with a ZETASIZER NANO ZS instrument (Malvern Panalytical, Worcestershire, UK) at 25 °C. The *ζ* value for each investigated sample was calculated considering the electrophoretic mobility (*μ*) of the particles by using the Smoluchowski relationship:

(3) ζ=ημ/κ for βα≫1

where η is the viscosity and *κ* represents the dielectric constant of the medium. *β* and *α* are the Debye–Hückel parameter and the particle radius, respectively.

Each measurement was performed three times, and the average value was considered.

The *rheological tests* were performed by using a MCR 302 rheometer (Anton Paar GmbH, Graz, Austria) (plane-plane geometry, diameter of 25 mm) at 25 °C. In order to limit the water evaporation, a solvent trap cover (Malvern Panalytical, Worcestershire, UK) was used. The flow curves were determined at pH values of 4 and 7.4 by applying a shear rate ($\dot{\gamma })$ from 10^−1^ 1/s to 10^3^ 1/s.

*ATR-FTIR spectra* were recorded on an FTIR Bruker Vertex 70 Spectrophotometer (Bruker Corporation, Billerica, MA, USA) in the wavenumber range of 4000–600 cm^−1^, with 32 scans and a resolution of 4 cm^−1^. The solid samples were grounded with potassium bromide powder and compressed into a disc for analysis. At pHs 2 and 7.4, the spectra were performed on the precipitated complexes. The solid from the mixture at pH = 4 was obtained by the evaporation of water. All solids were dried in an oven at 50 °C for a day. The spectra were processed using OPUS 6.5 software provided by Bruker Corporation.

### 3.4. Preparation of Structures for Simulation

The 3D structure of Lys was downloaded in .pdb format (PDB ID: 1REX) from the RCSB Protein Data Bank (https://www.rcsb.org (accessed on 3 February 2022)), and the water molecules were removed using Biovia Discovery Studio Visualizer v16.1.0.15350 (BIOVIA; Dassault Systèmes: San Diego, CA, USA; https://discover.3ds.com/discovery-studio-visualizer-download (accessed on 31 March 2021)). Lys structures at the desired pH (2, 4, and 7.4) were prepared on the PlayMolecule platform (https://www.playmolecule.com (accessed on 15 July 2023)) [69], and then they were optimized using Swiss PDB Viewer 4.1.0 software (http://www.expasy.org/spdbv/ (accessed on 15 July 2023)) [70].

The structures of PAA and PVA, with 7 and 4 structural units, respectively, were drawn in Avogadro 1.2.0 (https://avogadro.cc/releases/avogadro_120 (accessed on 14 July 2023)) [71]. The number of structural units has been selected considering the computer resources and the ratio between the number of structural units of PAA and PVA used in experimental measurements (Units_PAA_/Units_PVA_ = 6250/3773 = 1.7).

For the docking of PAA to Lys, the 3D structures of PAA in the ionized state corresponding to the pH of 2, 4, and 7.4 were used [72]. Therefore, at pH = 2, all groups are non-ionized (COOH) (the degree of ionization is α^−^ = 0%); at pH = 4, only one carboxylic group is ionized (α^−^ = 10%); and at pH = 7.4, all COOH groups are in an ionized state (COO^−^) (α^−^ = 100%). The geometry optimization of PAA and PVA 3D structures was performed in Avogadro 1.2.0 using the UFF force field with a steepest descent algorithm, and the optimized structures were saved as .pdb files.

### 3.5. Molecular Docking

Molecular docking was performed using PyRx-Python Prescription 0.8 with AutoDock Vina 1.1.2 as a docking engine and a Lamarckian Genetic Algorithm as a scoring function. (https://pyrx.sourceforge.io (accessed on 2 June 2021)) [73]. Lys molecule was imposed as macromolecule in rigid condition while ligands molecules were set as flexible molecules. The grid box parameters were defined individually for each pH to enclose the active sites previously provided by the CASTp (Computed Atlas of Surface Topography of Protein; http://sts.bioengr.uic.edu/castp/ (accessed on 15 July 2023)) [74] online server. All molecules were converted to .pdbqt form for docking by using PyRx-Python Prescription 0.8 software. For all dockings, the parameters were set as default, and only the exhaustiveness was changed to 32. The conformations with the most favorable energy were selected, and the interactions between Lys and PAA/PVA (hydrogen bonds, electrostatic interactions, hydrophobic interactions, etc.) on their 3D and 2D forms were analyzed with Discovery Studio Visualizer v16.1.0.15350.

## 4. Conclusions

The exploration of the interactions within the PAA–PVA–Lys complex in aqueous environments with varying pH values has provided valuable insights into the fundamental dynamics of this ternary system. The investigation revealed that the presence of PAA in the system significantly influences the coacervation zone, shifting the critical pH_1_ to higher values while leaving the critical pH_2_ unchanged. Moreover, this study elucidated the influence of Lys concentration on the coacervation zone, extending it with increasing Lys concentration. Zeta potential measurements highlighted the optimal stability of the PAA–PVA–Lys mixture within the pH range of 4.5–8. Beyond pH 9, the system becomes unstable, emphasizing the importance of maintaining the appropriate pH range for stability. Rheological analysis, specifically zero shear viscosity, exhibited deviations from additivity at both investigated pHs. The identification of maximum hydrodynamic volume at specific PAA weight fractions (0.4 and 0.5 for pHs 4 and 7.4, respectively) suggests the presence of critical points influencing the rheological properties of the system. Molecular dynamics simulations further emphasized the binding affinities within the system, revealing that the PAA–Lys complex is more stable than the PVA–Lys complex. The most stable PAA–Lys complex formed at pH = 2, with a binding affinity of −7.1 kcal/mol, attributed to stronger hydrogen bonds and less significant repulsive interactions between PAA and the protein. Considering published data [68] and our experimental and theoretical data, it can be concluded that PAA potentially acts as a bridge between PVA and Lys. The ability to modulate interactions through pH adjustments and PAA/PVA ratios presents opportunities for designing advanced materials with controlled properties. Overall, the insights gained from this study contribute to the fundamental understanding of complex formation and pave the way for innovations in biomedical and industrial applications of the PAA–PVA–Lys system.

## Figures and Tables

**Table 1:** The components of the Amide I band of Lys, extracted by curve–fitting, and the corresponding attributions.

Vibration (cm^−1^)	Tentative Assignment
1595	guanidyl CN_3_H_5_^+^ in Arg residues
1603–1610	NH_2_ scissor, hydrated chains, extended
1614–1618	extended beta sheets,
1625	aggregate, intermolecular beta sheets
1634	beta sheets, parallel
1644–1649	random coils
1651–1655	alpha helix
1660–1670	glutamine + beta turns
1673–1676	beta turns

**Table 2:** Rheological parameters by applying the Carreau–Yasuda model (Equation (1)).

w_PAA_	pH = 4		pH = 7.4	
	$\eta_{0}$ (mPa·s)	*n*	$\eta_{0}$ (mPa·s)	*n*
0	10.6 ± 0.06	0.93 ± 0.01	10.9 ± 0.42	0.95 ± 0.02
0.20	31.1 ± 0.07	0.97 ± 0.001	54.9 ± 0.31	0.89 ± 0.002
0.40	111.3 ± 11.22	0.87 ± 0.05	180.9 ± 1.21	0.64 ± 0.06
0.50	53.7 *	0.99 *	394.7 ± 2.01	0.74 ± 0.01
0.60	16.1 *	0.99 *	338.6 ± 4.05	0.78 ± 0.06
0.80	8.62 *	0.99 *	398.5 ± 2.85	0.57 ± 0.14
0.90	87.5 ± 0.39	0.96 ± 0.06	420.2 ± 1.07	0.62 ± 0.02
1	122.6 ± 0.90	0.77 ± 0.07	461.1 ± 1.62	0.68 ± 0.02

* The errors are very small.

**Table 3:** CASTp results concerning the active site amino acids of Lys at various pHs.

pH = 2	pH = 4	pH = 7.4
LEU31 TRP34 SER36 ASN44 ASN46 THR52 TYR54 ILE56 PHE57 GLN58 ILE59 ASN60 TYR63 TRP64 VAL99 GLN104 ALA108 TRP109 VAL110 ALA111	LEU31 TRP34 SER36 ASN44 ASN46 ASP49 SER51 THR52 TYR54 ILE56 PHE57 GLN58 ILE59 ASN60 ARG62 TYR63 TRP64 VAL99 GLN104 ALA108 TRP109 VAL110 ALA11	GLU35 ASN46 ASP49 SER51 ASP53 GLN58 ILE59 ASN60 ARG62 TYR63 TRP64 VAL99 GLN104 ALA108 TRP109 VAL110 ALA11

**Table 4:** Details concerning the binding interactions of PAA docked to the active site of Lys.

pH	Binding	Favorable Bonds		Unfavorable Bonds
	Affinity (kcal/mol)	Classical Hydrogen (Bond Distance, Å)	Other Types (Bond Distance, Å)	Type (Bond Distance, Å)
2	−7.1	GLH35 (2.02) ASN46 (2.04) ASN46 (2.52) ASH49 (2.53) ASN60 (2.44) ASN60 (2.48) TYR63 (2.39) TRP64 (2.60) VAL110 (2.05)	–	donor–donor TRP64 (1.26) ALA111 (1.48)
4	−6.6	GLH35 (1.89) ASN46 (2.76) ASP49 (2.56) SER51 (2.63) ASH53 (2.31) GLN58 (2.75) ASN60 (2.33) ASN60 (2.47) ASN60 (2.63) TRP64 (2.20) TRP64 (2.63) VAL110 (2.99) ALA111 (2.10)	carbon hydrogen ILE59 (3.07)	negative–negative GLH35 (2.83) ASH53 (5.45)
7.4	−6.4	ASN46 (2.69) SER51 (2.70) ASN60 (2.42) ASN60 (2.59) TYR63 (2.63) TYR63 (1.98) TRP64 (2.38) TRP64 (2.75) GLN104 (2.08) TRP109 (2.96) VAL110 (2.24)	carbon hydrogen ILE59 (3.43) salt bridge ARG113 (2.36) pi–anion TYR63 (4.07)	negative–negative GLU35 (4.68) ASP53 (4.27) ASP53 (5.16)

**Table 5:** Details concerning the binding interactions of PVA docked to the active site of Lys.

pH	Binding	Favorable Bonds		Unfavorable Bonds
	Affinity (kcal/mol)	Classical Hydrogen (Bond Distance, Å)	Other Types (Bond Distance, Å)	Type (Bond Distance, Å)
2	−5.25	GLH35 (2.41) GLH35 (2.23) ASN60 (2.40) VAL110 (2.88) ALA111 (2.00)	pi–sigma TRP109 (3.75)	–
4	−5.3	ASN46 (2.57) SER51 (2.44) ASH53 (2.73) ASN60 (2.60) TRP109 (2.88)	pi–sigma TRP109 (3.63)	–
7.4	−5.3	SER51 (2.41) ASP53 (1.88) ASP53 (2.72) GLN58 (2.27) TRP109 (2.88)	pi–sigma TRP109 (3.63)	donor–donor ASN60 (1.73)

## Data Availability

Data are contained within the article and Supplementary Materials.

## Supplementary Materials

The following supporting information can be downloaded at: https://www.mdpi.com/article/10.3390/molecules29010208/s1, Figure S1: Spectral decomposition of the carbonyl stretching/Amide I region of: (a) PVA–PAA complex; (b) Lys; (c) S2 precipitate; (d) S4; (e) S7.4. Figure S2: Apparent viscosity, η, *versus* shear rate, $\dot{\gamma }$, for the PAA–PVA–Lys mixtures with *c_p_* = 2.5%, *c_Lys_* = 0.2%, and different weight fractions of PAA in PAA–PVA mixture at (a) pH = 4 and (b) pH = 7.5 at 25 °C. The lines represent the fitting according to the Carreau-Yasuda model.
