# Supplementary material for: Effect of pH on the Poly(acrylic acid)/Poly(vinyl alcohol)/Lysozyme Complexes Formation
Source: Molecules. 2023 Dec 29;29(1):208. doi: 10.3390/molecules29010208 (PMC10780248; doi:10.3390/molecules29010208)
Supplement: Supplementary file 1 [file molecules-29-00208-s001.zip › molecules-2780409-supplementary.pdf]

Supplementary Information for

Effect of pH on the Poly(Acrylic Acid)/Poly(Vinyl Alcohol)/Lysozyme Complexes Formation

Simona Morariu*, Mihaela Avadanei, Loredana Elena Nita

“Petru Poni” Institute of Macromolecular Chemistry, 41-A Grigore Ghica Voda Alley, 700487, Iasi, Romania

*Correspondence: smorariu@icmpp.ro

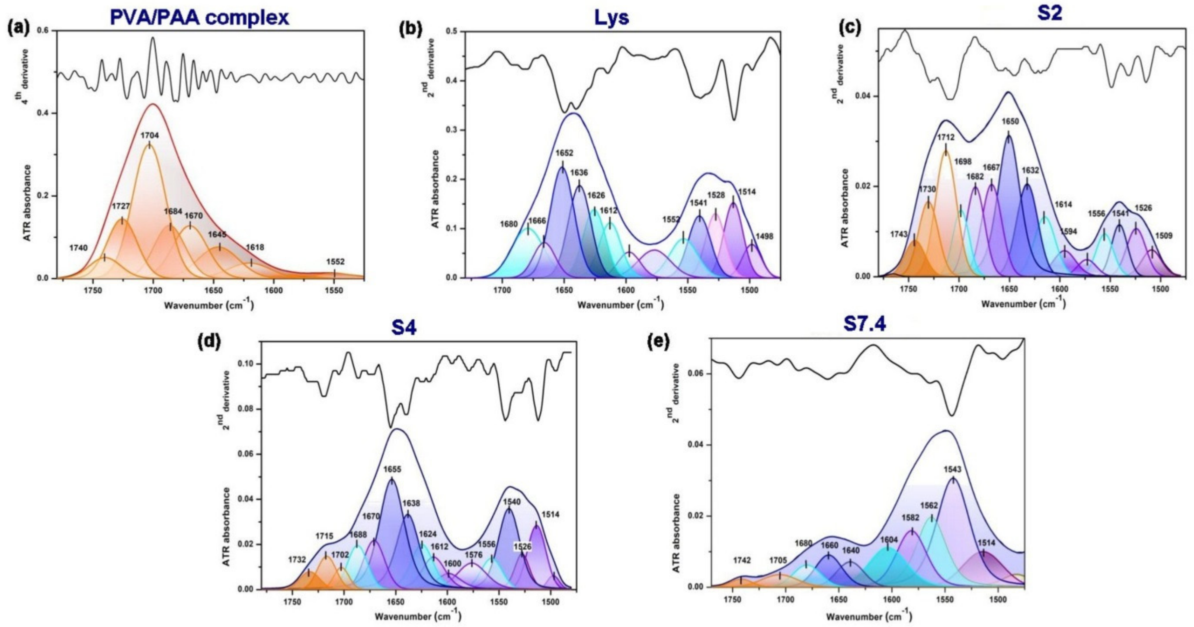

Figure S1. Spectral decomposition of the carbonyl stretching/Amide I region of: (a) PVA–PAA complex; (b) Lys; (c) S2 precipitate; (d) S4; (e) S7.4.

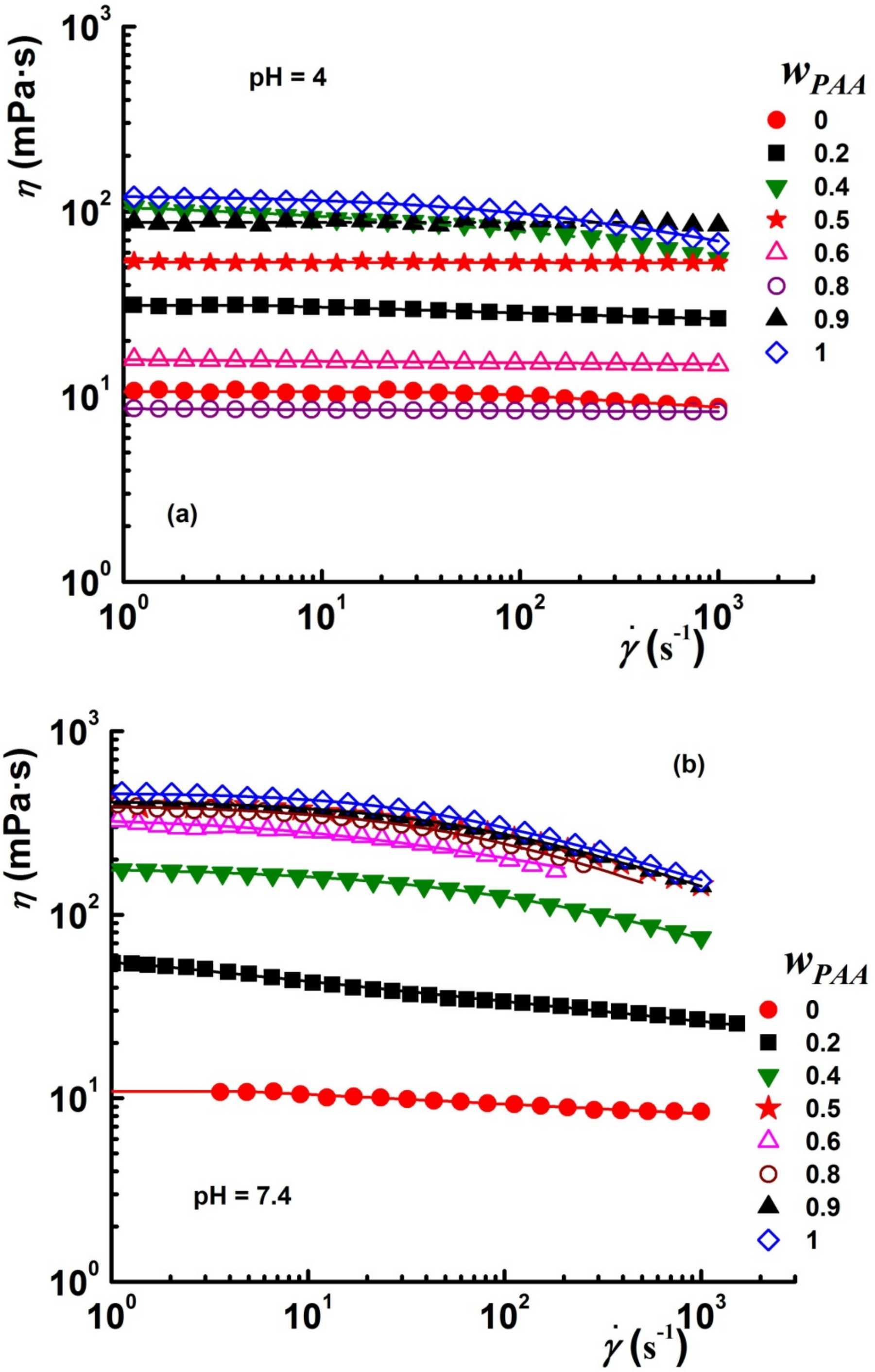

Figure S2. Apparent viscosity, $\boldsymbol{\eta}$, *versus* shear rate, $\dot{\boldsymbol{\gamma}}$, for the PAA–PVA–Lys mixtures with c_p = 2.5%, c_{Lys} = 0.2%, and different weight fractions of PAA in the PAA–PVA mixture at (a) pH = 4 and (b) pH = 7.5 at 25 °C. The lines represent the fitting according to the Carreau-Yasuda model.
